# Translation and Psychometric Validation of the German Version of the Iceland–Family Perceived Support Questionnaire (ICE-FPSQ): A Cross-Sectional Study

**DOI:** 10.1177/10748407241234262

**Published:** 2024-04-15

**Authors:** Katinka Freudiger, Lotte Verweij, Rahel Naef

**Affiliations:** 1 Zurich University of Applied Science, Winterthur, Switzerland; 2 University of Zurich, Switzerland; 3 University Hospital Zurich, Switzerland

**Keywords:** family nursing, perceived support, family, German translation, psychometric testing, intensive care unit, self-report measure

## Abstract

Supporting families experiencing critical illness through family interventions is essential to ease illness burden, enable family management, and reduce their risk for adverse health. Thus far, there is no validated German instrument to measure the perceived support families receive from nurses. We translated the 14-item Iceland–Family Perceived Support Questionnaire (ICE-FPSQ) and tested its psychometric properties with 77 family members of intensive care patients. Compared with the original instrument, the construct validity of the German ICE-FPSQ (FPSQ-G) showed unstable results with a partially divergent structure, most likely caused by the limited sample size. The first two principal components explained 61% of the overall variance and a good internal consistency with a Cronbach’s alpha of .92. The FPSQ-G is a promising instrument to measure family members’ perceptions of the support they received from nurses in the acute critical care setting but requires further validation.

## Introduction

Family members of patients admitted to intensive care units (ICUs) are exposed to psychological burden (Alfheim et al., 2018; Eggenberger & Nelms, 2007) and experience a wide range of different mental and physical health challenges during and after the critical illness (Minton et al., 2019; van Beusekom et al., 2016), resulting in increased levels of stress, uncertainty, depression, and anxiety (Alfheim et al., 2019; Davidson et al., 2012; Haines et al., 2015). This set of symptoms has been defined as family ICU syndrome (FICUS) or post–intensive care syndrome–family (PICS-F; Davidson et al., 2012; Netzer & Sullivan, 2014).

Nurses have a significant influence on how families perceive and experience the care provided to them (Eggenberger & Nelms, 2007; Mackie et al., 2019). It is argued that nurses are ideally situated to address families’ needs because of their constant presence and nursing mandate (Al-Mutair et al., 2013; Au et al., 2019; Kaakinen et al., 2018). Best practice recommendations for family interventions in adult ICUs point to the need to offer interventions, such as family presence in the ICU, emotional and practical support, structured communication, and the use of consultations with professionals and members of the ICU team (Davidson et al., 2017; Harvey & Davidson, 2011; Kaakinen et al., 2018). Research has shown promising results on the benefit of family interventions in ICU (Kentish-Barnes et al., 2022; Kiwanuka et al., 2022; Naef et al., 2020; Naef, von Felten, et al., 2021; White et al., 2018).

To systematically test the effectiveness of family interventions, several instruments have been developed that measure family functioning and family health (Kaakinen et al., 2018; Sawin, 2016). One of the most widely used instruments for the evaluation of family interventions in nursing is the Iceland–Family Perceived Support Questionnaire (ICE-FPSQ). This instrument measures the perceived support from nurses in the acute–critical care setting and was developed in Iceland (Sveinbjarnardottir et al., 2012) based on the Calgary Family Assessment and Intervention Models (CFAM/CFIM; Wright & Leahey, 2013). The ICE-FPSQ was validated in family members of medical, surgical, pediatric, geriatric, and psychiatric patients at an Icelandic hospital and showed good psychometric properties (Sveinbjarnardottir et al., 2012). The questionnaire has so far been translated (a) into Swedish and validated in parents with children suffering from heart disease (Bruce et al., 2016); (b) into Dutch, validated in three public hospitals in family members of patients with a chronic disease (Konradsen et al., 2018); (c) into Portuguese, validated in parents of children and adolescents with chronic conditions (Lemos et al., 2022), and (d) into isiZulu, validated on three emergency departments (Emmamally & Brysiewicz, 2019). Furthermore, the ICE-FPSQ has been translated into English and Norwegian, but it has not been psychometrically tested yet (Aass et al., 2022; Sveinbjarnardottir et al., 2012).

The ICE-FPSQ has been used in several studies to test family interventions (Gisladottir et al., 2017; Gusdal et al., 2018; Rosenstrøm et al., 2023; Svavarsdottir et al., 2012; Sveinbjarnardottir et al., 2013). For example, Sveinbjarnardottir and colleagues (2013) achieved significantly higher levels of perceived support in family members of patients with psychiatric disorders receiving family interventions than in those receiving standard care. Furthermore, the ICE-FPSQ was used to examine the effect of nursing educational interventions on family members, for instance, in the study by Beierwaltes and colleagues (2020), where a significant improvement on seven out of 14 items was found. Other studies have used the questionnaire to identify differences between patient and family perceptions of support (Aass et al., 2022; Dieperink et al., 2018; Gusdal et al., 2018). For example, family members of patients suffering from mental illness rated perceived support significantly lower, with a total mean sum score of 30.7, than patients with a total mean sum score of 40.3 (Aass et al., 2022). The questionnaire can also be used, as Emmamally and Brysiewicz (2019) did, to assess the current level of care provided to families and for international comparison. Another possible application of the ICE-FPSQ was shown by Eggenberger and Sanders (2016) who used the questionnaire to assess the need for more family support. Overall, the ICE-FPSQ is a widely applied instrument within family nursing and is being used in various cultures and care settings, such as acute and chronic care, emergency department, pediatrics, and psychiatric care.

To our knowledge, there is no validated instrument available to measure the perceived support to families as a system by nurses for the German-speaking population. To close this gap, we have translated and subsequently tested the psychometric properties of the ICE-FPSQ in family members of critically ill persons admitted to Swiss ICUs. In addition, we aimed to evaluate family members’ perceived support from Swiss ICU nurses.

## Method

### Design

A correlative cross-sectional design with principal component analysis (PCA) was used to test for construct validity of the ICE-FPSQ after translating the instrument into German.

### Setting and Samples

The study was carried out on six ICUs of a University Hospital in the German-speaking part of Switzerland, which hosts around 4,500 admissions per year. The ICUs utilize primary nursing (Mattila et al., 2014) and offer nursing interventions, such as structural interprofessional meetings, daily phone calls to family members, and the provision of information materials. During the COVID-19 pandemic, visits were restricted to one single visitor per patient for a maximum of 1 hour per day. Individual exceptions were possible.

The study population included family members (i.e., patients and close others) of patients admitted to an adult ICU between July 2021 and March 2022 and hospitalized in the ICU for ≥48 hours. Family members were eligible if they had at least one interaction with an ICU nurse, were ≥18 years, and returned the questionnaire ≥4 months after ICU discharge to counteract potential recall bias. If several family members per patient fulfilled the inclusion criteria, they were considered eligible. Based on Bühner’s (2011) recommendation for the minimum sample size in PCA, we aimed for at least 200 completed questionnaires.

### Recruitment

Family members were recruited either by invitation of ICU nurses or by flyers/posters in the waiting areas of the ICUs from November 2021 to March 2022. In addition, family members were contacted through patients who received the study documents by mail. Patients admitted from July to December 2021 were identified by searching the hospital’s electronic database. Patients who had not given their general consent for use of their clinical data for research purposes were excluded. In total, 315 patients were contacted. Regardless of how they were recruited, family members could choose between a paper-based questionnaire (prepaid return envelope included) and an electronic version based on an online data capture system (REDCap; https://www.project-redcap.org). Family members were instructed to complete the questionnaire between the end of the patient’s ICU stay to 4 months after discharge. Submitting the completed questionnaire to the research team was considered as informed consent for participation.

### The Instrument: ICE-FPSQ

Based on the CFAM/CFIMs (Shajan & Snell, 2019; Wright & Leahey, 2013), Sveinbjarnardottir and colleagues (2012) developed the ICE-FPSQ questionnaire to evaluate family members’ perceived support from professional caregivers in the acute-critical setting. The ICE-FPSQ includes five items on the perceived “cognitive support” and nine items on the perceived “emotional support” and was psychometrically tested in family members of patients from several specialities (e.g., medical, surgical, and geriatric) of an Icelandic hospital. The internal consistency of the original ICE-FPSQ was measured using Cronbach’s alpha coefficients of reliability and showed a value of .953 for the entire scale, .874 for the cognitive, and .937 for the emotional subscale, which indicates a high consistency (Sveinbjarnardottir et al., 2012). Each item of the ICE-FPSQ can be rated by a 5-point Likert-type scale with responses ranging from 1 (*almost never*) to 5 (*almost always*). The maximum overall score is 70, that is, a maximum of 25 points for the perceived “cognitive support” and a maximum of 45 points for the perceived “emotional support.” The higher the total score, the better the family member perceived support of the professional caregivers (Sveinbjarnardottir et al., 2012). However, there is no description of what score must be given for a good or insufficient support from nurses or other health professionals.

### Translation

The ICE-FPSQ was translated from English to German using a forward and backward translation procedure following Sousa and Rojjanasrirat (2011). First, two independent translators who were native speakers of German translated the instrument from English to German. Then, the translators and the research team discussed the results, resolved discrepancies, and agreed on a first German version of the instrument. Next, the first German version was translated back into English by two other translators whose mother tongue is English. Finally, a consensus meeting was held with one translator each from the forward and backward translation process and the research team to agree on a final German version (FPSQ-G). All four translators had different professional backgrounds.

### Content Validity

The content validity of the FPSQ-G was tested by using the Content Validity Index (CVI; Polit & Beck, 2017; Sousa & Rojjanasrirat, 2011) with five purposively sampled family care experts. The Item-CVI (I-CVI) values ranged from 0.8 to 1, while the Scale-CVI/Average (S-CVI/Ave) value was 0.96. These results demonstrated good content validity for the German version of the ICE-FPSQ (Polit et al., 2007; Schwanda, 2016). In addition, the family care experts were asked to rate the comprehensibility of the individual German items on a scale of 0 points (*not at all*) to 10 points (*very much*). The overall comprehensibility was rated at 8.6 points (*SD* = 0.86, min–max = 6.8–9.6). Written feedback of the experts on comprehensibility exclusively concerned the content of the original items and not the translation itself; thus, no changes were necessary.

Subsequently, a pretest was conducted with five purposively sampled family members of ICU patients to evaluate the comprehensibility of the FPSQ-G. All participants confirmed the comprehensibility of the questionnaire.

### Criterion Validity

To test criterion validity of the FPSQ-G, two measurements were used. First, we added a single question, similar to the original ICE-FPSQ, asking the family members to rank the overall quality of support from 0 points (*not satisfied*) to 10 points (*really satisfied*). The question was placed after the last item of the FPSQ-G.

Second, we included the German version of the FS-ICU 24R, an established instrument to measure family members’ satisfaction with the quality of care in the ICU (Stricker et al., 2007; van den Broek et al., 2015; Wall et al., 2007). The instrument has two subscales, “satisfaction with care” (14 items) and “satisfaction with involvement in decision-making” (10 items). Each item is rated on a 5-point Likert-type scale from 1 (*very dissatisfied*) to 5 (*completely satisfied*). The maximum score of 100 represents maximum satisfaction with care. The instrument showed good internal consistency with Cronbach’s alpha >.87, with no floor or ceiling effect (Stricker et al., 2007).

### Demographics and Other Information

In addition, demographics and characteristics of family members (age, sex, highest educational qualification, employment status, type of relationship, cohabitation with patient, and previous experience as family member on the ICU) and of their ill family member (age, sex, living situation, reason for ICU admission, and length of ICU stay), and frequency and type of contact between family member and nurses were obtained. Regarding the COVID-19-related restricted visiting hours, we evaluated family members’ perspective on the restricted visiting hours. Family members were asked how they perceived the COVID-19-related restricted visiting hours and whether they experienced them as emotionally stressful. Both questions could be rated on a 5-point Likert-type scale (*very strong*—*not at all*).

### Data Analysis

Statistical analysis was performed in R Version 4.1.2. (R Core Team, 2016). The significance level was set at 5%.

The data from the ICE-FPSQ were checked for missings. Two questionnaires were discarded because they had ≥30% missing answers (Hair et al., 1998; Wirtz, 2004). Three questionnaires, which had less than 30% missing answers, were imputed using the statistical multiple imputation by chained equation (MICE) package in R (Buuren & Groothuis-Oudshoorn, 2011).

For demographic and characteristic data, we calculated frequencies and percentages, as well as means and standard deviations for the metric variables. Using the Kaiser–Meyer–Olkin Index (KMO index) and the Bartlett test, we tested whether the data were suitable for a PCA (Bühner, 2011; Wolff & Bacher, 2010). To be able to interpret the results of the PCA, the data were orthogonally rotated at the level of the factor analysis using the Varimax rotation to determine how the individual item is weighted to the extracted factors (Moosbrugger & Kelava, 2012; Polit & Beck, 2004). The number of relevant principal components (PCs) was selected using the Kaiser criterion and the Scree test visualized as a screeplot (Moosbrugger & Kelava, 2012). The internal consistency was calculated using Cronbach’s alpha. Criterion validity was evaluated by conducting a Spearman’s correlation test of the overall score of the FPSQ-G and the single question of quality of perceived support. Furthermore, the overall score of the FPSQ-G was also tested for correlation with the overall score of the FS-ICU 24R using a Spearman’s correlation test.

In addition, we also investigated whether the FPSQ-G is dependent on the age or sex of the participants by examining the data using an unpaired Wilcoxon test. For analysis, participants were divided into two groups (younger, older) based on the mean age of 54.2 years. Subsequently, this binary variable was included in our analysis instead of the continuous age variable.

### Ethical Consideration

The responsible ethics committee reviewed the study and waived the need for approval based on Swiss law. Online completion and return of the paper questionnaire by mail was considered as “informed consent.”

## Results

### Sample

**Figure 1.**
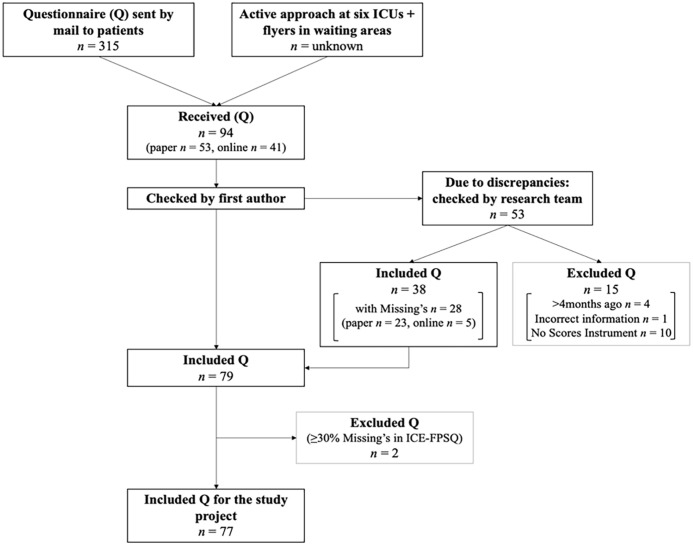
Data Cleaning Process. Note. ICU = intensive care unit.

In total, 315 patients were invited by mail, and an unknown number of family members were invited through a flyer or an active approach by the nurses on the six ICUs. At the end of data collection, the research team received 94 questionnaires, of which 77 questionnaires could be included for analysis after the data cleaning process (for more details, see Figure 1).

Most participants (*n* = 46, 61%) filled out the questionnaire 3 to 4 months after ICU discharge, 21% (*n* = 16) between ICU discharge and 3 months, and 18% (*n* = 14) during ICU stay. Family members were on average aged 54.2 years (*SD* = 15.22, *n* = 76), and 61% (*n* = 47) of them were women. The family member’s reported relationship to the patient was partner (55%, *n* = 42), child (19%, *n* = 15), parent (14%, *n* = 11), sibling (7%, *n* = 5), or friend (5%, *n* = 4). The respective patients were on average aged 58.4 years (*SD* = 15.8, *n* = 75), and 40% (*n* = 31) of them were women. The mean length of ICU stay was 18.1 days (*SD* = 26.62, *n* = 75). Patients were admitted due to an emergency (47%, *n* = 41), a transfer from another ward or ICU (34%, *n* = 30), or after a planned operation or intervention (19%, *n* = 16). Half of the family members (53%, *n* = 41) had contact with nurses once a day, 38% (*n* = 29) several times a day, 8% (*n* = 6) several times a week, and 1% less often (*n* = 1). Most of the family members reported that conversations with nurses mainly happened face-to-face (65%, *n* = 45) followed by phone calls (35%, *n* = 24). A total of 38% (*n* = 29) of the family members stated that they already had experience as a family member in an ICU. Details on demographics and characteristics are shown in Tables 1 and 2.

**Table 1.** Demographics and Characteristics of Family Members.

Age in years, *M* ± *SD; Mdn*; min–max; *n* = 76	54.2 ± 15.22; 56; 23–87
Sex, *n* (%); *n* = 77 Male Female	30 (39) 47 (61)
Highest educational qualification, *n* (%); *n* = 77	
Primary education Secondary education (diploma, certificates) Higher education (bachelor’s/master’s degree) Other	29 (38) 23 (30) 17 (22) 8 (10)
Employment status, *n* (%); *n* = 77 Employed Unemployed Retired	50 (65) 8 (10) 19 (25)
Relationship to patient, *n* (%); *n* = 77 Partner Daughter or son Mother or father Sister or brother Friend	42 (55) 15 (19) 11 (14) 5 (7) 4 (5)
Cohabiting with the patient, *n* (%); *n* = 77	
Yes No If no, how often do you have contact with your family member, *n* (%); *n* = 27	48 (62) 29 (38)
Several times a week Once a week Once a month	19 (70) 6 (22) 2 (8)
Previous experience as family member on the ICU, *n* (%); *n* = 77	
Yes No	29 (38) 48 (62)
Restricted visiting hours experienced, *n* (%); *n* = 77	
Yes No Don’t know	36 (47) 34 (44) 7 (9)
Visiting hours perceived as restrictive, *n* (%); *n* = 36	
Very strong Rather strong Undecided Rather not Not at all	8 (22) 2 (6) 4 (11) 18 (50) 4 (11)
Emotional stress due to restricted visiting hours, *n* (%); *n* = 36	
Very strong Rather strong Undecided Rather not Not at all	6 (17) 5 (14) 6 (17) 12 (33) 7 (19)

*Note*. For continuous data, *M* ± *SD, Mdn*, min–max, and sample size (*n*) are shown. Absolute numbers (*n*) and percentages (%) are shown for discrete data.

**Table 2.** Demographics and Characteristics of Patients Reported by Family Members.

Age in years, *M* ± *SD; Mdn*; min–max; *n* = 75	58.43 ± 15.8; 60; 20–90
Sex, *n* (%); *n* = 77 Male Female	46 (60) 31 (40)
Patient lives, *n* (%); *n* = 77 Alone Partner With family (>2 persons) With someone else	12 (16) 51 (66) 11 (14) 3 (4)
Reason for ICU admission, *n* (%); *n* = 77 Emergency Transferred from another unit/ICU Planned after surgery/intervention	41 (47) 30 (34) 16 (19)
Length of ICU stay in days, *M* ± *SD; Mdn*; min–max; *n* = 75	18.07 ± 26.62; 12; 2–210

*Note*. For continuous data *M* ± *SD, Mdn*, min–max, and sample size (*n*) are shown. Absolute numbers (*n*) and percentages (%) are shown for discrete data.

### Construct Validity

The KMO test showed a value of .889, which can be considered meritorious and thus meets a first requirement for factor analysis (Bühner, 2011; Kaiser, 1974). The Bartlett test revealed strong correlations of variables, χ^2^(91) = 638.83, *p* < .001, which is also a requirement for factor analysis (Wolff & Bacher, 2010). Hence, we could apply a PCA to our data.

**Figure 2.**
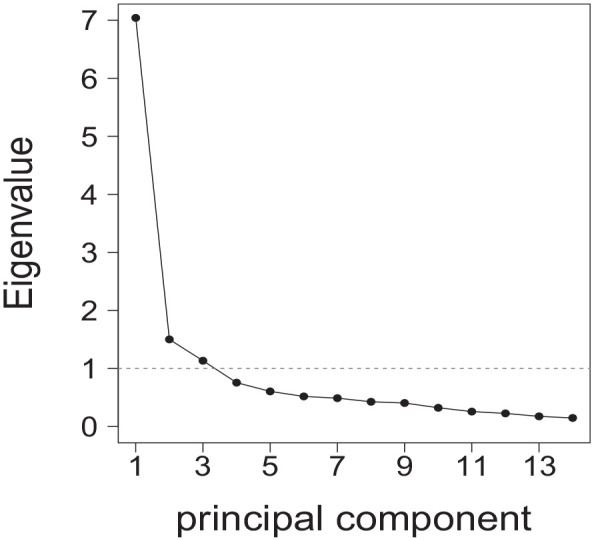
Screeplot of Eigenvalues. *Note*. The horizontal dashed line indicates the threshold of eigenvalue = 1. PCs scoring an eigenvalue ≥1 fulfill the Kaiser criterion and, therefore, could be included in further analyses.

The eigenvalues, visualized in a screeplot (see Figure 2), indicated a solution including three PCs in line with the Kaiser criterion to include PCs with eigenvalues above 1 (Moosbrugger & Kelava, 2012). PC1 and PC2 had eigenvalues from 7.04 and 1.50, respectively. PC3 showed an eigenvalue of 1.13. Hence, an additional two-factorial solution according to the original study was used.

The degree of cumulative variance for two PCs together was moderate with 61%. PC1 explained 34%, while PC2 explained 27% of the cumulative variance. In contrast to the original instrument, it is noticeable that Item 1 (offered us information and their professional opinion) loaded on PC1 instead of PC2 and the Items 6 (offered us family meetings) and 12 (encouraged my family to take a respite from caregiving) loaded on PC2 instead of PC1 as in the original version. Furthermore, Item 10 (drawn out our family strengths) loaded similarly high on PC1 and PC2 compared with the original instrument. This means that PC2, as a cognitive support component, has six items instead of five, and PC1, as an emotional support component, has eight items instead of nine in the German version, compared with the original instrument.

The PC loadings of items, except for the first item (.47), were higher than .50 (min–max = .47–.85; more details in Table 3). The commonality is defined as the proportion of the total variance of the individual variables that is explained by the factors (Moosbrugger & Kelava, 2012). With a mean of .61 (*SD* = .13, min–max = .22–.72), the commonality in our study was high in relation to the whole instrument. The cognitive subscale scored a mean of .63 (*SD* = .1, min–max = .42–.72), while the emotional subscale scored a mean of .59 (*SD* = .16, min–max = .22–.72).

**Table 3.** PC Loadings and Scoring of Family Members (n = 77) on the FPSQ-G.

Item in FPSQ-G	PC1 (emotional)	PC2 (cognitive)
The nurses on the unit have. . .		
1. Offered us information and their professional opinion	.47	<.30
2. Provided accessible and easy-to-read literature about the health problem	<.30	.75
3. Informed my family about the resources available in the community that have proven to be helpful for families in similar situations	<.30	.85
4. Provided ideas, information, and thoughts in a manner that enabled us to learn from them and reflect on them	.41	.73
5. Emphasized the use of family rituals to promote family members’ health	.51	.67
6. Offered us family meetings	.44	.64
7. Helped family members recognize that our emotional response is valid and helped us to validate and/or normalize family members’ emotional response	.71	.32
8. Encouraged my family to become involved with the health care team in the care of our family member and have offered us caregiver support	.70	.34
9. Encouraged family members to share their illness narratives—not only stories of illnesses and suffering but also stories of strength and resilience	.75	.40
10. Drawn out our family strengths	.56	.52
11. Helped family members understand how our emotional response is related to the family member’s illness	.71	.34
12. Encouraged my family to take a respite from caregiving	.44	.52
13. Been aware of the impact family members can have on one another, on the patient’s well-being, and on the illness itself	.84	<.30
14. Looked for the family’s strengths and opportunities to commend family members when their strengths have been revealed	.77	.30

*Note*. The second column shows the loadings on PC1 (emotional support component) and the third column PC2 (cognitive support component), respectively. The primary loadings are displayed in black, while the secondary loadings are displayed in gray.

### Internal Consistency

Cronbach’s alpha for the whole instrument with its 14 items was excellent with .92. It scored satisfying (.86) for the cognitive support and excellent (.90) for emotional support.

### Family Members Perceived Support

The overall score of the FPSQ-G averaged 43.8 points (*SD* = 14.07, min–max = 18–70, *n* = 77) out of a maximum possible score of 70 points. For the cognitive subscale (six items) the mean was 16.43 points (*SD* = 6.86, min–max = 6–30, *n* = 77) out of 30 points and for the emotional subscale (eight items) 27.38 points (*SD* = 8.4, min–max = 11–40, *n* = 77) out of 40 points. The response frequencies per item are presented in Table 4. The family members rated the quality of support from the nurses with an average of 8.5 points (*SD* = 1.83, min–max = 2–10, *n* = 68) out of a maximum of 10 points.

**Table 4.** Distribution Among Response Categories on the Items of the FPSQ-G (n = 77).

Item in FPSQ-G	Results *n* (%)					
	Almost never (1)	Rarely (2)	Sometimes (3)	Usually (4)	Almost Always (5)	*M, SD*
The nurses on the unit have. . .						
1. Offered us information and their professional opinion	0 (0)	0 (0)	9 (12)	17 (22)	51 (66)	4.55 ± 0.7
2. Provided accessible and easy-to-read literature about the health problem	26 (34)	9 (12)	14 (18)	12 (15)	16 (21)	2.78 ± 1.56
3. Informed my family about the resources available in the community that have proven to be helpful for families in similar situations	32 (41)	9 (12)	19 (25)	7 (9)	10 (13)	2.4 ± 1.44
4. Provided ideas, information, and thoughts in a manner that enabled us to learn from them and reflect on them	18 (23)	15 (20)	23 (30)	10 (13)	11 (14)	2.75 ± 1.34
5. Emphasized the use of family rituals to promote family members’ health	26 (34)	10 (13)	12 (16)	15 (19)	14 (18)	2.75 ± 1.54
6. Offered us family meetings	24 (31)	6 (8)	13 (17)	14 (18)	20 (26)	3 ± 1.61
7. Helped family members recognize that our emotional response is valid and helped us to validate and/or normalize family members’ emotional response	12 (15)	5 (7)	5 (7)	18 (23)	37 (48)	3.82 ± 1.48
8. Encouraged my family to become involved with the health care team in the care of our family member and have offered us caregiver support	14 (18)	5 (6)	13 (17)	22 (29)	23 (30)	3.45 ± 1.45
9. Encouraged family members to share their illness narratives—not only stories of illnesses and suffering but also stories of strength and resilience	21 (27)	8 (10)	12 (16)	21 (27)	15 (20)	3.01 ± 1.51
10. Drawn out our family strengths	25 (32)	11 (14)	15 (20)	14 (18)	12 (16)	2.7 ± 1.48
11. Helped family members understand how our emotional response is related to the family member’s illness	22 (29)	10 (13)	15 (19)	16 (21)	14 (18)	2.87 ± 1.49
12. Encouraged my family to take a respite from caregiving	25 (32)	6 (8)	20 (26)	16 (21)	10 (13)	2.74 ± 1.44
13. Been aware of the impact family members can have on one another, on the patient’s well-being, and on the illness itself	6 (8)	6 (8)	10 (13)	24 (31)	31 (40)	3.88 ± 1.25
14. Looked for the family’s strengths and opportunities to commend family members when their strengths have been revealed	17 (22)	11 (14)	17 (22)	12 (16)	20 (26)	3.09 ± 1.5

*Note*. The values in the answer categories represent those leading to the total score of the FPSQ-G. The second to sixth columns show the response frequencies with *n* and %. The last column contains the average scoring of the individual items, reported as *M* ± *SD*. FPSQ-G = German version of Iceland–Family Perceived Support Questionnaire.

### Criterion Validity

The average overall score of FS-ICU 24R was 84.04 points (*SD* = 16.26, min–max = 23–118, *n* = 73) out of a maximum of 100 points. The total scores of the FPSQ-G and the FS-ICU 24R correlated moderately (Spearman’s *r_s_* = .438, *p* < .001, *n* = 73). Furthermore, the single question (quality of support from nurses, scored from 0 to 10 points) showed a significant and strong correlation with the total score of the FPSQ-G (Spearman’s *r_s_* = .679,*p* < . 001, *n* = 68).

### Effect of Sex and Age

The scores of the FPSQ-G are neither depended on family members’ sex (Wilcoxon, *W* = 913.5, *p* = .334, *n_w_* = 42, *n_m_* = 28) nor on age (Wilcoxon, *W* = 705.5, *p* = .09, *n_y_* = 27, *n_o_* = 42).

## Discussion

The psychometric properties of the FPSQ-G as tested with 77 family members of patients treated in intensive care showed good psychometric properties, except for construct validity. Construct validity indicated a two-component solution despite meritorious KMO and a satisfactory Bartlett test, which showed differences in the loading of individual items compared with the original. Three items did not load on the same PC as the original, whereas another item loaded similarly high on both PCs. However, the communality concerning the whole instrument was very high. Internal consistency showed good results for the entire instrument and the two subscales. Criterion validity was confirmed by a significant correlation between both, the FPSQ-G and the FS-ICU 24R, as well as the FPSQ-G and the single question assessing quality of perceived support. Furthermore, we found that age and sex were no confounders on the total score of the FPSQ-G. The family members rated the perceived support from the nursing staff during the patient’s ICU stay on average 43.8 out of 70 maximum points.

Validity was determined by the construct validity, criterion validity, and internal consistency of the FPSQ-G. The results of the PCA for construct validity were unsatisfactory. Here, the diverging results with the original instrument are particularly noticeable and could be influenced mainly by the low sample size of 77 participants. In addition, the different PC loadings of Items 1, 6, and 12, and the similar loading of item 10, might also be explained by the formulation of these items, which is open for interpretation by the participants. The cognitive subscale is described in the original instrument as support consisting of informing and educating families to help them cope with the experience of illness (Sveinbjarnardottir et al., 2012). The emotional subscale is defined as support of family members in coping and managing emotional distress (Sveinbjarnardottir et al., 2012). However, Item 1, which captures the prevalence of information available and the professional opinion of the caregiver, loads on the emotional PC and not on the cognitive PC. We hypothesize that making this item more concrete regarding the kind of information provided by nurses (e.g., leaflets about certain diseases) might make a difference in the loading of the item. In its current version, the item is open to the participants’ interpretation of the extensive term “Information.” Item 6 asks whether the nurses offered family meetings. In our study, the item loads on the cognitive instead of the emotional PC compared with the original instrument. Again, we suspect that if family meetings were more clearly defined in the item, this might result in loading on the emotional PC. In our study, family members might have rated interprofessional conversations (which have the goal of providing information and involving them in decision-making and are routinely provided at the ICUs of the university hospital) instead of family meetings with nurses aiming at helping them cope with emotional distress. A possible solution would entail making items less open to interpretation by using more concrete and narrower terms or providing participants with a short definition or example of what is being referred to (Streiner et al., 2015). Item 12 asks whether the family was encouraged by nurses to take a respite from caregiving and should load on the emotional subscale. We hypothesize that this may have happened because family members might have interpreted the nurses’ intention as not wanting to deal with them and not that of a genuine concern for their well-being. At least in some instances, this interpretation might have even been correct as a feeling of anxiety and being uncomfortable around family members has been reported as a barrier to successful family involvement in the literature (Hetland et al., 2018; Naef, Brysiewicz, et al., 2021). The unexpected loading of Item 10 on both PCs might be explained, after all, by the translation. The German verb “aufzeigen” has a much stronger informative and cognitive connotation than the original English verb “to draw out” and is more similar to “to show/demonstrate.” However, the limited sample size of 77 complete questionnaires likely has had the most influence on the unstable results and the differences in loading of the first, sixth, and 12th items and the unclear primary loading of the 10th item because a sample size of 200 participants is recommended for PCA (Bühner, 2011; Hair et al., 2019).

PC3 was discarded due to its low eigenvalue and following the two-component solution of the original instrument. Different procedures to identify the PCs are described in the literature. However, it is often challenging to choose the main PCs with the highest explained variance while excluding PCs that explain only a small variance of the total variance. In our study, PC1 and PC2 explained 61% of the variance, which is generally considered a satisfactory result in social science (Hair et al., 2019). The individual loads of the items, which load on the same PC as the original, correspond approximately to similar values as those of the Swedish PCA (Bruce et al., 2016; Sveinbjarnardottir et al., 2012). However, it should be noted that Item 5 of the Swedish version of the ICE-FPSQ loaded similarly on both PCs, which was not the case with the FPSQ-G (Bruce et al., 2016).

The internal consistency for the overall scale measured by Cronbach’s alpha was nearly the same as in the original version with .961 (Sveinbjarnardottir et al., 2012). The cognitive scale had a difference of minus .02 in the German version compared with the original version (.881); the Cronbach’s alpha for the emotional subscale in the German version is .5 lower compared with the original version (.95; Sveinbjarnardottir et al., 2012). The Swedish and Danish translations of the ICE-FPSQ also show higher internal consistency in the emotional compared with the cognitive component. However, their absolute values are more in line with those in the original version than ours (Bruce et al., 2016; Konradsen et al., 2018). These comparisons show that the German translation of the ICE-FPSQ is on par with the original and the Danish and Swedish translations.

In the literature, criterion validity is an important quality criterion for the measurement properties of questionnaires in the field of health status (Terwee et al., 2007). Our results exceed the gold standard of at least .70 (Terwee et al., 2007). Sex and age could be excluded as confounders on the total score. Compared with our results, the Danish study described a significant influence of age on the total scores and the scores of the two subscales of the ICE-FPSQ (Konradsen et al., 2018).

During the data cleaning, it was noticed that some participants added handwritten *never* instead of *almost never* to the Likert-type Scale of the ICE-FPSQ or left a comment that they could not answer the questions because the answer possibility they wanted to choose did not exist or they had not experienced the specific intervention at all. Although family interventions might be implemented in theory, they are often still not applied in daily practice due to various reasons (Hetland et al., 2017; Naef, Brysiewicz, et al., 2021). Perhaps in a revised version, an additional response option, such as *never*, could be added to counteract a possible bias.

On average, family members of ICU patients rated the perceived support from nursing staff with 43.8 points out of a maximum of 70 points on the FPSQ-G and the quality was rated with 8.5 points on average on a scale of 0 to 10. These results suggest that family members did not always perceive the supportive interventions measured by the FPSQ-G. We cannot exclude that the perception of the family members in our research project was negatively influenced by the COVID-19 pandemic. Study results from research projects on flexible visiting hours in ICUs even before the pandemic indicated that flexible visiting hours had a positive impact on family members and patient satisfaction and reduced rates of delirium and anxiety (Junior et al., 2018; Mitchell & Aitken, 2017). Therefore, the more restrictive visiting hours caused by the COVID-19 pandemic may have negatively influenced family members’ experiences, although participants’ perception of these restrictions was less negative than expected (see Table 1). This might be explained by the fact that most participants cannot compare the visiting regulations with pre-pandemic times. However, the restricted visiting hours were only one aspect of the pandemic; more serious was the impact of the increased workload of the ICU staff and the scarcity of resources, which likely had a significant impact particularly on family interventions. Naef and Monteverde (2021) found that the pandemic and its restrictive measures in hospitals led to a shift away from institutionalized, evidence-based, and family-centered interventions to a focus on infection prevention. Alternative solutions to family integration under the challenging pandemic situation led to an additional burden at the physical, psychological, and ethical levels for health workers (Naef & Monteverde, 2021; Saghafi et al., 2022).

Recruiting family members in the ICU was challenging. The response rate for the paper-based questionnaires was unexpectedly low at around 30%. Active recruitment of family members by ICU nursing staff could not be carried out to the extent hoped, mainly due to limited staff resources. This caused the most significant limitation of our study with a small sample size of 77 participants. Furthermore, multicenter data collection would exclude possible institutional influences on the results (e.g., barriers to family care interventions) and could strengthen the robustness of the validation results. For future purposes, we would recommend conducting a confirmatory factor analysis (CFA) following the PCA. Especially, regarding the discrepancies between the loadings in our study and those of the original instrument, the possible reasons could be statistically determined by a CFA procedure, which a pure PCA does not provide (Moosbrugger & Kelava, 2012). For reproducibility, the data could be checked by the intraclass correlation coefficient (ICC), which tests how robust the research results are and thus represents another quality feature (Terwee et al., 2007).

## Conclusion

The FPSQ-G showed a two-component solution based on PCA in the setting of family members of ICU patients. Due to the limited sample size, our findings should be interpreted with caution. Still, the FPSQ-G shows that it is a promising instrument to capture family members’ perceptions of the support they received from nurses in the acute–critical care setting of ICUs.
